# Lungworms of Non-Ruminant Terrestrial Mammals and Humans in Iran

**DOI:** 10.3390/pathogens12060759

**Published:** 2023-05-25

**Authors:** Sina Mohtasebi, Alireza Sazmand, Salman Zafari, Guilherme G. Verocai, Domenico Otranto

**Affiliations:** 1 Faculty of Veterinary Medicine, University of Calgary, Calgary, AB T2N 4Z6, Canada; sina.mohtasebi@ucalgary.ca; 2 Department of Pathobiology, Faculty of Veterinary Science, Bu-Ali Sina University, Hamedan 6517658978, Iran; domenico.otranto@uniba.it; 3 Department of Medical Parasitology and Mycology, School of Medicine, Hamadan University of Medical Sciences, Hamadan 6517838736, Iran; s.zafari@edu.umsha.ac.ir; 4 Department of Veterinary Pathobiology, School of Veterinary Medicine and Biomedical Sciences, Texas A&M University, College Station, TX 77843, USA; 5 Department of Veterinary Medicine, University of Bari, 70010 Bari, Italy

**Keywords:** lungworms, nematoda, non-ruminants, verminous pneumonia, wildlife, Iran

## Abstract

With over 300 terrestrial and aquatic mammalian species, Iran is considered a country with an ample mastofauna. Although many studies have assessed the distribution of gastrointestinal helminth parasites in animals and humans in Iran, lungworms have not received adequate attention. Following a previous article in which we reviewed the diversity and prevalence of lungworm infections in pastoral and wild ruminants of Iran, this report compiles the available scientific information about the occurrence of lungworms in non-ruminant mammals and humans from 1980 to 2022 to provide insights into the epidemiology of these infections. International and national scientific databases were searched, and twenty-six articles in peer-reviewed journals, one conference paper, and one D.V.M. thesis were included in the study. In total, 10 species belonging to seven genera, including *Dictyocaulus*, *Deraiophoronema*, *Protostrongylus*, *Crenosoma*, *Eucoleus*, *Aelurostrongylus*, and *Metastrongylus*, were reported in the respiratory tract or feces of humans, domestic animals (i.e., camels, equids, dogs, and cats), and wildlife species (i.e., hedgehogs, wild boars, and hares). Most of the studies (22/28) were performed using post-mortem examinations. The overall prevalence of respiratory nematode infection varied according to animal species in camels (14.83%), equids (13.31%), dogs (5%), wild boars (45.66%), hedgehogs (42.57%), and hares (1.6%). In addition, pulmonary capillariasis caused by *Eucoleus aerophilus* was reported in a 9 year old child. The prevalence of lungworm species in domestic camels, equids, and dogs, combined with a lack of labeled anthelmintic products, supports the need to improve our understanding of these important nematode parasites and inform the development of sustainable control strategies. From a zoo and wildlife medicine point of view, there is a shortage of information about the presence and prevalence of lungworm infections in the majority of mammalian species, pending epidemiological studies that integrate classical parasitology and molecular methods.

## 1. Introduction

Parasitic infections occur in almost every animal community and play significant roles in the mortality and morbidity of the hosts they inhabit [1]. Many helminths parasitize the lungs and respiratory tracts of many domestic and wild species globally [2,3]. The detrimental economic impacts of these pathogens on farm animals have urged scientists to do research on them, whereas the ecology and fauna of lungworms in many wild species are still unknown [4].

The vast geographical expanses of Iran have provided suitable habitats for a variety of both domesticated and wild animals [5]. Indeed, Iran is a country with an ample mammal fauna, with approximately 192 terrestrial and 16 aquatic species of mammals, including iconic species such as the Asiatic cheetah *Acinonyx jubatus* (Schreber, 1775), the Persian leopard *Panthera pardus saxicolor* (Pocock, 1927), the Mesopotamian fallow deer *Dama dama* subsp. *mesopotamica* (Brooke, 1875), and the Asiatic wild ass *Equus hemionus* (Pallas, 1775) [6,7]. As previously reviewed, seven species of lungworms belonging to four genera, i.e., *Dictyocaulus filaria* (Rudolphi, 1809), *Dictyocaulus viviparus* (Bloch, 1782), *Dictyocaulus eckerti* (Skrjabin, 1931), *Protostrongylus rufescens* (Leuckart, 1865), *Protostrongylus raillietti* (Schulz, Orlow and Kutass, 1933), *Muellerius capillaris* (Müller, 1889), and *Cystocaulus ocreatus* (Railliet and Henry, 1907) have been reported in Iran, in domestic sheep, goats, cattle, water buffaloes and wild ruminants, such as urials, *Ovis orientalis* (Gmelin, 1774); wild goats, *Capra hircus* subsp. *Aegagrus* (Erxleben, 1777); goitered gazelles, *Gazella subgutturosa* (Güldenstaedt, 1780); roe deer, *Capreolus capreolus* (Linnaeus, 1758); and red deer, *Cervus elaphus* (Linnaeus, 1758) [8]. However, very little information is available regarding the lungworms of non-ruminant terrestrial mammals in the country. 

In this article, we reviewed the available information about the status of nematode infections in the respiratory tract of equids, camels, wild boars, hedgehogs, hares, dogs, cats, and humans in Iran.

## 2. Materials and Methods

The search period for articles was from October 2018 to February 2023. In order to find related articles and studies, scientific resources and the Google Scholar search engine were used alongside IranDoc, Civilica, PubMed, and CAB Direct websites singularly and in combination, using English, Persian, and French language search strings “lungworm (s), mammals, carnivores, domestic, wild, wildlife; plus, common and scientific Latin names of the hosts and lungworms”. Searches were conducted in Persian (Farsi) and English languages. After the collection of the documents, the validity and accuracy of the studies were screened for clarity, originality, objectivity, sampling methods, diagnostic methods, and percentage of evaluation studies. An Excel file of the above information was prepared. Data were extracted as follows: study area, species of infected animal, species of helminths, prevalence of lungworms infecting animals, year of study, number of animals studied, and source of inspection (feces or carcass).

## 3. Results and Discussion

**Figure 1:**
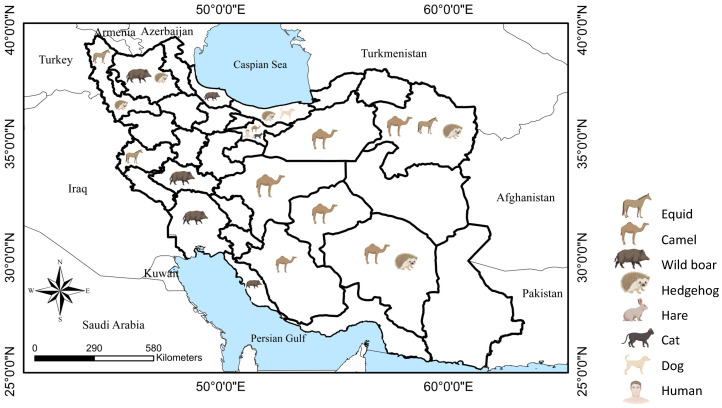
Map of Iran showing the distribution of reports of non-ruminant terrestrial mammals and humans.

The collected documents were written in Persian and English, and all the reports were published after 1980. Among 31 publications, three studies were excluded since they did not match the validity and accuracy evaluation criteria. In the twenty-eight studies included in this review (i.e., twenty-six articles in peer-reviewed articles; twenty-three in English and three in Persian, one conference paper in Persian, and one D.V.M. thesis in Persian with an English abstract), 10 species of lungworms belonging to seven genera, e.g., *Dictyocaulus* (Railliet and Henry, 1907), *Deraiophoronema* (Romanovitch, 1916), *Protostrongylus* (Kamensky, 1905)*, Crenosoma* (Molin, 1861)*, Eucoleus* (Dujardin, 1845)*, Aelurostrongylus* (Cameron, 1927), and *Metastrongylus* (Molin, 1861) have been reported in the respiratory system or feces of different domestic and wild terrestrial mammals namely, horse *Equus caballus* (Linnaeus, 1758), donkey *Equus asinus* (Linnaeus, 1758), mule (*Equus asinus* × *Equus caballus*), one-humped camel *Camelus dromedarius* (Linnaeus, 1758), dog *Canis lupus* subsp. *Familiaris* (Linnaeus, 1758), cat *Felis catus* (Linnaeus, 1758), Southern white-breasted hedgehog *Erinaceus concolor* (Martin, 1838), long-eared hedgehog *Hemiechinus auritus* (Gmelin, 1770), wild boar *Sus scrofa* (Linnaeus, 1758), and cape hare *Lepus capensis* (Linnaeus, 1758) (Figure 1). Most of the studies (22/28) were performed using post-mortem examination of animal carcasses.

### 3.1. Equid Lungworms

In Iran, horses, donkeys, and their hybrids, including male horse × female donkey (hinnies), female horse × male donkey (mules), are widely distributed across the country. Three publications reported eggs and larvae of *Dictyocaulus arnfieldi* (Cobbold, 1884) in horses, donkeys, and mules in Iran, including one research article, one case report, and one D.V.M. thesis [9,10,11]. So far, adult nematodes have not been isolated, and all of the conducted studies employed fecal flotation and the Baermann technique, with an overall prevalence of *D. arnfieldi* of 10.04% (50/498) in horses, 31.81% (21/66) in donkeys, and 24.32% (9/37) in mules, consistent with the literature stating that donkeys are more suitable definitive hosts of *D. arnfieldi* than horses as they can tolerate large numbers of parasites with few clinical signs of respiratory disease and are considered natural reservoirs [12]. Of note, the first-stage larvae of *D. arnfieldi* were recovered from the feces of two horses in a zoo in Mashhad, northeast of Iran, with a history of being co-housed with donkeys for a short time [9] (Table 1). However, in addition to domesticated equids, the IUCN red list includes endangered species [13]. The Persian onager, *Equus hemionus* subsp. *Onager* (Boddaert, 1785), is native to the deserts of Iran. No studies are available about *Dictyocaulus* sp. infection in onager populations, while in the only parasitological study available, *Gasterophilus pecorum* (Fabricius, 1794) and *Habronema muscae* (Carter, 1861) were isolated from the stomach of one dead animal [14]. Furthermore, although donkeys seem to play a more substantial role in the maintenance of this parasite than other horses [15], there is no information about their hybrids: hinnies and mules, which are commonly found in Iran and can be the subject of future research.

### 3.2. Nematodes of the Respiratory Tract of Camels

Nine publications reported lungworm infection in one-humped camels, including eight peer-reviewed articles [16,17,18,19,20,21,22,23] and one conference paper [24]. Approximately 152,000 Old World camels (OWCs), including one-humped (dromedary) and two-humped (Bactrian) camels (only 100–300 individuals), are scattered throughout 21 of the 31 Iranian provinces [25,26]. In Iran, *D. filaria* has been isolated from the lungs of examined dromedaries [18,27], implying cross-infection with ruminants. As the measurements have not been reported, it is not possible to assess the correct identification and differentiation from *Dictyocaulus cameli* (Boev, 1951). In general, compared with other livestock species, relatively few reports are available on the status of lungworm infections in camels, and a study on thirty-four camels in Kazakhstan over seven decades ago reported the infection of camels with *D. cameli* and a few *D. filaria* [28]. Although in the revision of the genus it was concluded that *D. cameli* is a valid species [29], there is not much known, especially about the epidemiology and molecular characterization of this nematode. So, the collection of *Dictyocaulus* from dromedaries and Bactrians in camel-rearing areas of the world through a multi-state study is advocated in order to increase our knowledge about this scantly known species. However, due to the fact that the life cycle of *Dictyocaulus* species requires moist conditions for the survival of the larvae, lungworm infection should not be a severe issue for dromedaries living in hot and dry climates. In addition to bronchial system-dwelling lungworms *D. cameli*, *D. filaria*, and *D. viviparus* [26], mature worms of the camel-specific filarioid nematode *Deraiophoronema evansi* (Lewis, 1882), commonly known as *Dipetalonema evansi*, inhabit various organs, including the pulmonary arteries, heart, testicles, epididymis, and spermatic cord [22,30]. In this article, we only considered documents reporting adult *D. evansi* worms from the respiratory system of dromedaries and pathological lesions associated with them; however, several other articles reported observation and/or molecular detection of the helminth microfilariae in the blood of 0.88–46.7% of dromedaries [26]. Studies reported in Table 2 examined the presence and pathology of lungworms postmortem. Overall, the prevalence of lungworm infections in tested camels in Iran was 16.9% (142/840) for *D. evansi* and 11.1% (10/90) for *Dictyocaulus*, reported as *D. filaria*. In neighboring Turkey, the prevalence of *D. viviparus* in wrestling hybrid camels (hybrids of female dromedary camels and male Bactrian camels) was 5.5% [31]. However, so far, this helminth species has not been reported in Iranian camels. Although there have been many coproscopy-based studies in Iran, the Baermann technique has not been applied in research to diagnose the L1 in feces. Hence, it is recommended to perform larvoscopy along with regular coproscopy in future studies.

### 3.3. Suid Lungworms

In six peer-reviewed articles, lungworm infections were reported in wild boars in Iran [32,33,34,35,36,37]. According to the Islamic Republic of Iran’s laws, which are based on Islamic precepts, retail and consumption of pork are illegal in the country, and subsequently, no industrial pig farming has been present since 1978. However, wild boars are widespread and occasionally get killed, mainly when they invade private farms and destroy crops. In Iran, three *Metastrongylus* species, including *Metastrongylus apri* (Gmelin, 1780) (syn. *Metastrongylus elongatus*), *Metastrongylus pudendotectus* (Wostokow, 1905) (syn. *Metastrongylus brevivaginatus*)*,* and *Metastrongylus salmi* (Gedoelst, 1923), have been reported in wild boars (Table 3). In the studies above, the overall prevalence of lungworm infection in tested wild boars was 45.66% (58/127) using both fecal flotation (prevalence 23.62%; 30/127) and post-mortem examination (prevalence 76.38%; 97/127). Similarly, the same species composition and prevalence as in Iran were reported in Turkey, with *M. apri* (59%) being the most prevalent, followed by *M. salmi* and *M. pudendotectus* (both 52%) [38]. Suids are known to become infected with eight lungworm species, including *M. apri*, *M. pudendotectus*, and *M. salmi*, which have been reported from Iran, as well as *M. confusus*, *M. asymmetricus*, *M. madagascariensis*, *M. pulmonalis*, and *M. tschiauricus* [39,40], so understanding the possible presence of other *Metastrongylus* species in Iran needs future studies. In addition, from a public health point of view, human infection with suid lungworms rarely occurs, and five cases, four with *M. elongatus* and one with *M. salmi*, have been reported in Ecuador so far [39,41,42]. So, the role of *Metastrongylus* species as zoonotic pathogens must not be overlooked, and the environment and wildlife diseases sectors and researchers must be vigilant about the surveillance and occurrence of these helminths in swine alongside other known zoonotic parasites such as *Ascaris suum* (Goeze, 1782); *Trichinella spiralis* (Owen, 1835); and *Balantidium coli* (Malmsten, 1857). 

### 3.4. Hedgehog Lungworms

In six peer-reviewed articles, the presence, prevalence, and pathology of lungworms were reported in hedgehogs in Iran [43,44,45,46,47,48]. Four species of hedgehogs, including the southern white-breasted hedgehog *E. concolor*, the long-eared hedgehog *H. auratus*, the Brandt’s hedgehog *Paraechinus hypomelas* (Brandt, 1836), and the desert hedgehog *Paraechinus aethiopicus* (Ehrenberg, 1832), occur in Iran [7]. Unfortunately, in some previous parasitology reports from Iran, species-level identification of hedgehogs was not performed correctly (discussed in Khodadadi et al., 2021 [49]); however, in this review article, we cross-matched the reports with the natural geographical habitats of each hedgehog species. In Iran, infection of southern white-breasted hedgehogs and long-eared hedgehogs with *Crenosoma striatum* (Zeder, 1800) has been recorded in Mazandaran, Kerman, Razavi Khorasan, and East Azerbaijan provinces (Figure 1). Furthermore, the southern white-breasted hedgehog has been reported to host *Eucoleus aerophilus* (Creplin, 1839) in the northwestern province of West Azarbaijan, which neighbors Turkey [48]. Overall, 38.61% (39/101) and 9.09% (4/44) of examined animals were found infected with *C. striatum* and *E. aerophilus,* respectively, after post-mortem examination (Table 4). These two nematodes were the only species reported in a study [50] on road-killed hedgehogs in neighboring Turkey, with *C. striatum* and *E. aerophilus* found in 55.5% and 22.2% of animals, respectively. Considering that *E. aerophilus* is potentially zoonotic and there has been a rising interest in keeping hedgehogs as exotic pets globally [51], it is suggested that Iranian veterinarians be watchful of the diagnostic, treatment, and management of parasitic infections in these playful animals.

### 3.5. Lagomorph Lungworms

In the only documentation on lungworm infections in wild lagomorphs of Iran, *Protostrongylus raillieti* (Schulz, Orlow, and Kutass, 1933) was reported in 4 out of 420 (1.6%) cape hares collected from 24 provinces [52]. This nematode usually infects caprine ruminants such as *Ovis aries* subsp. *Orientalis* (Gmelin, 1774) [53]. In total, seven lungworm species belonging to the genus *Protostrongylus* (Nematoda, Protostrongylidae) are observed in lagomorphs worldwide: *Protostrongylus pulmonalis* (Frölich, 1802), *Protostrongylus terminalis* (Passerini, 1884); *Protostrongylus kamenskyi* (Schulz, 1930); *Protostrongylus cuniculorum* (Joyeux and Gaud, 1946); *Protostrongylus tauricus* (Schutz and Kadenatsii, 1949); *Protostrongylus boughtoni* (Goble and Dougherty, 1943); and *Protostrongylus oryctolagi* (Babo, 1955) [54]. However, according to a previous historical review on the morphological identification of *Protostrongylus* species isolated from Lagomorpha, there have been several confusions on the characterizations, for instance between *P. terminalis* and other species such as *P. pulmonalis* and *P. rufescens*, and also between *P. cuniculorum* and *P. rufescens* [55]. Similarly, *Protostrongylus* specimens isolated from *L. capensis* in Iran could potentially have been misidentified. Hence, the collection and reassessment of novel materials would be necessary to integrate morphological and molecular characterization. Furthermore, three other wild lagomorph species, namely the European hare *Lepus europaeus* (Pallas, 1778); the Tolai hare *Lepus tolai* (Pallas, 1778); and the Afghan pika *Ochotona rufescens* (Gray, 1842), occur in Iran [6], but despite their wide geographic distribution in the country [56], there have been no studies about their lungworm fauna. Therefore, further research on Lagomorpha in Iran is required to fill these many gaps.

### 3.6. Dog Lungworms

Only a single study reported eggs of the trichuroid nematode *E. aerophilus* during the fecal examination of 5 out of 100 domestic dogs in Mazandaran province [57], making our knowledge of the status of canine lungworms in Iran fragmentary. However, the eggs of *E. aerophilus* and *Eucoleus boehmi* (Supperer, 1953) are similar in morphological characteristics [58], so it is strongly recommended to perform multicenter epidemiological studies to shed light on the *Eucoleus* fauna of dogs in the country.

In this single Iranian study, fecal specimens were concentrated by the formalin-ether sedimentation method and flotation technique in a saturated zinc chloride solution [57]. Since cystic echinococcosis is hyper-endemic in the country and dogs are the main final hosts of *Echinococcus granulosus* (Batsch, 1786) sensu lato [59], handling and examining the canine fecal specimens require special care, and hence the Baermann technique for recovery of potentially present L1 larvae of metastrongyloid lungworms was not performed. Subsequently, information about the epidemiology of canid lungworms has remained limited in the country, though the infection can be diagnosed by other techniques such as imaging, and by examination of biological samples other than feces, including bronchoalveolar lavage, nasal secretions, and vomit [60]. 

Although *E. aerophilus* has been reported, it is known that dogs become infected mainly with *Oslerus osleri* (Cobbold, 1876) but also with *Filaroides hirthi* (Georgi and Anderson, 1975); *Filaroides milksi* (Whitlock, 1956); and *Crenosoma vulpis* (Dujardin, 1844), which are normally parasites of wild canids such as foxes, coyotes, and wolves [61]. However, despite the indispensable epidemiological roles of wild canids in lungworm infection of domestic dog populations, there is an absolute lack of information about the possible presence of lungworms in the various wild canid species widely present in Iran, such as the golden jackal *Canis aureus* (Linnaeus, 1758), the red fox *Vulpes vulpes* (Linnaeus, 1758), and the grey wolf *Canis lupus* (Linnaeus, 1758). It is worth mentioning that in recent years, the rocketing population of stray dogs has become a veterinary and public health concern in Iran. Hence, to raise scientific knowledge regarding the risk of lungworm infections in owned dogs, it is suggested to search for the lungworms infecting stray dog populations and wild canids in different regions of the country. 

There has been an increasing habit of keeping dogs as pets in Iran in the past two decades. Considering that *E. aerophilus* is known to be potentially zoonotic (discussed in Section 3.8), it is strongly advisable to routinely include lungworms in the differential diagnosis of cardiorespiratory diseases in dogs.

### 3.7. Cat Lungworm

Only one paper in Iran has reported the presence of *Aelurostrongylus abstrusus* (Railliet, 1898) larvae circulating in the blood of a stray cat from Tehran [62]. In that study, researchers applied the Knott’s test to determine the presence of microfilariae in filarioid nematodes; therefore, the finding of *A. abstrusus* L1 was considered incidental [62]. Cats are the usual hosts of *A. abstrusus*, and feline aelurostrongylosis has been reported in different geographical regions with different prevalence rates [63], but there is limited information about West Asian countries. In a study in Qatar, aelurostrongylosis was reported in 7.5% of feline fecal samples [64], and the infection of cats in Iraq and Turkey was documented as case reports [65,66]. While *A. abstrusus* is considered to be the most prevalent lungworm of cats, other respiratory nematodes such as *Troglostrongylus* spp. (Vevers, 1923), *Oslerus rostratus* (Gerichter, 1945), and *E. aerophilus* have been reported from cats [60,67], none of which have been reported from Iran. However, a recent epidemiological study in Israel described *Troglostrongylus brevior* (Gerichter, 1949) as the dominant lungworm infecting feral cats in Jerusalem, Israel [68], suggesting that *T. brevior* is present in other Middle Eastern countries, including Iran. In the absence of information about lungworm infections in Iranian wild felids, including the Asiatic cheetah *A. jubatus*, the Persian leopard *P. saxicolor*, the caracal *Caracal caracal* (Schreber, 1776), Pallas’s cat *Otocolobus manul* (Pallas, 1776), and the Caucasian lynx *Lynx lynx dinniki* (Satunin, 1915), the wildcat *Felis silvestris ornate* (Gray, 1832), the jungle cat *Felis chaus* (Schreber, 1777), and the sand cat *Felis margarita* (Loche, 1858), conducting studies on these larger cats would be extremely informative considering that domestic and wild felids share several lungworm species, such as *A. abstrusus*, *E. aerophilus*, and *T. brevior* [60,69], and possibly additional lungworm species.

### 3.8. Human Lungworm

In Iran, *E. aerophilus* was once recorded in a 9 year old boy with severe asthmatic symptoms, a productive cough, and moderate eosinophilia, whose symptoms were alleviated by prescribing diethylcarbamazine, thiabendazole, and steroids [70]. Human respiratory capillariasis caused by *E. aerophilus* is rare, with only 12 reported cases globally [71,72]. While the infection is more common in canine and feline medicine, one major issue in assessing the status of this complication is the lack of implementation of adequate diagnostic methods, and therefore many cases might go underdiagnosed [71]. In addition, human capillariosis is characterized by non-specific clinical signs such as fever, bronchitis, coughing, hemoptysis, and dyspnea and, importantly, may induce relevant damage resembling bronchial carcinoma [71]. Epidemiological information from the examination of dogs and cats throughout the country may help physicians assess the risk of human infections in each area.

## 4. Conclusions, Remarks, and Challenges for Future Research

The present review describes the considerable diversity of nematode species infecting the respiratory tract of wild and domesticated non-ruminant mammals in Iran, including humans. Although the relevance of nematodes infecting the respiratory tract of various mammalian hosts has been largely neglected when compared to other parasitic nematodes, they are considered important pathogens, causing significant complications in domestic and wild animals worldwide. In our previous review article, we highlighted the status quo of lungworms in pastoral livestock systems in Iran [8], and herein we noted the diversity of lungworms in non-ruminant mammals and humans. As previously mentioned, environmental conditions such as seasonal changes, rainfall, and temperature may directly influence the occurrence of lungworm infection in different host species and their epidemiology. Moreover, it should be noted that climate change, loss of biodiversity, animal trade, and a lack of large-scale surveillance can play key roles in fluctuations observed in the prevalence and diversity of species in different regions. The diversity of lungworms, their host associations, and prevalence have not received enough attention in the scientific community of Iran, and complementary studies are needed to describe the status of respiratory nematode species in Iran, especially in wild hosts. In the current study, it was shown that the majority of cases had been analyzed in post-mortem examinations, but it should be considered that finding carcasses of wild animals is difficult, so administering standard parasitological diagnostic tools on fresh biological specimens as well as developing robust and novel techniques to study older environmental samples could improve the diagnosis of parasites. Additionally, the use of classical and molecular techniques could boost the accuracy of diagnosis, especially in morphologically similar species.

## Figures and Tables

**Table 1:** Lungworm *Dictyocaulus arnfieldi* in equids from Iran according to geographical area.

Host	% Prevalence	Number of Examined Host	Geographical Region	Study Area	Year of Study	References
Horse	Case report	2	Eastern provinces	Mashhad	2008	[9]
Horse	1.4	140	Western provinces	Kermanshah	Not stated	[11]
Horse	13.2	356	Northwestern provinces	Urmia	2014–2016	[10]
Donkey	31.8	66		Urmia	2014–2016	[10]
Mule	24.3	37		Urmia	2014–2016	[10]

All of the studies used the Baermann technique, except one [11] that employed fecal flotation for the diagnosis.

**Table 2:** Nematodes affecting the respiratory tract of camels (*Camelus dromedarius*) in Iran according to geographic area and nematode species.

Nematode Species	% Prevalence	Number of Examined Host	Geographical Region	Study Area	Year of Study	References
*Dictyocaulus filaria*	3.3	30	Northern provinces	Tehran	Not stated	[16]
*Deraiophoronema evansi*	9.9	30		Tehran	Not stated	[16]
Verminous pneumonia (*Dictyocaulus viviparus*)	1.0	100		Semnan	2011	[17]
*Dictyocaulus filaria*	10.0	60	Southern provinces	Kerman	Not stated	[18]
*Deraiophoronema evansi*	17.5	40		Shiraz	Not stated	[19]
*Deraiophoronema evansi*	8.4	309		Kerman	2009–2010	[20]
*Deraiophoronema evansi*	27.0	100		Fars and Yazd	Not stated	[21]
*Deraiophoronema evansi*	2.8	144		Yazd, Najaf-Abad, and Rafsanjan	Not stated	[22]
*Deraiophoronema evansi*	34.5	197	Eastern provinces	Mashhad, Afghanistan, and Pakistan	2003–2006	[23]
*Deraiophoronema evansi*	18.0	50		Mashhad	2008–2009	[24]

All these studies used post-mortem examinations of carcasses at an abattoir.

**Table 3:** Lungworms of wild boars (*Sus scrofa*) in Iran according to geographical area and nematode species.

Nematode Species	% Prevalence	Number of Examined Host	Geographical Region	Study Area	Year of Study	References
*Metastrongylus apri*	Case report	1	Northern provinces	Talesh	2013	[32]
*Metastrongylus apri*	41.6	12	Western provinces	Lorestan	2000–2001	[33]
*Metastrongylus pudendotectus*	16.6	12		Lorestan	2000–2001	[33]
*Metastrongylus salmi*	8.3	12		Lorestan	2000–2001	[33]
*Metastrongylus apri*	16.7	30	Northwestern provinces	East Azarbaijan	2015	[34]
*Metastrongylus* sp.	Case report	2	Southwestern provinces	Khuzestan	Not stated	[35]
*Metastrongylus* sp.	68.0	25		Bushehr	2013	[36]
*Metastrongylus apri*	16.0	57	National parks and protected regions of Iran		Not stated	[37]
*Metastrongylus pudendotectus*	14.0	57			Not stated	[37]
*Metastrongylus salmi*	14.0	57			Not stated	[37]

All the studies except one [34], which employed direct smears and fecal flotation for the diagnosis, studied the carcasses postmortem.

**Table 4:** Lungworms of hedgehogs in Iran according to geographical area and nematode species.

Nematode Species	% Prevalence	Host	Number of Examined Host	Geographical Region	Study Area	Year of Study	References
*Crenosoma striatum*	20.0	*Erinaceus concolor*	10	Northern provinces	Babol	2011	[43]
*Crenosoma striatum*	37.5	*Erinaceus concolor*	8		Mazandaran	2010–2011	[44]
*Crenosoma striatum*	4.3	*Hemiechinus auritus*	23	Eastern provinces	Mashhad	2010–2014	[45]
*Crenosoma striatum*	100	*Hemiechinus auritus*	6	Southern provinces	Kerman	Not stated	[46]
*Crenosoma striatum*	61.0	*Erinaceus concolor*	44	Northwestern provinces	Urmia	2009–2011	[48]
*Crenosoma striatum*	Not stated	Not stated	10		Urmia	2012	[47]
*Eucoleus aerophilus*	9.0	*Erinaceus concolor*	44		Urmia	2009–2011	[48]

All animals were studied postmortem.

## Data Availability

Not applicable.
